# Polymorphisms in Arsenic(+III Oxidation State) Methyltransferase (*AS3MT*) Predict Gene Expression of *AS3MT* as Well as Arsenic Metabolism

**DOI:** 10.1289/ehp.1002471

**Published:** 2010-09-24

**Authors:** Karin Engström, Marie Vahter, Simona Jurkovic Mlakar, Gabriela Concha, Barbro Nermell, Rubhana Raqib, Alejandro Cardozo, Karin Broberg

**Affiliations:** 1 Department of Laboratory Medicine, Section of Occupational and Environmental Medicine, Lund University, Lund, Sweden; 2 Institute of Environmental Medicine, Section for Metals and Health, Karolinska Institutet, Stockholm, Sweden; 3 University Medical Centre Ljubljana, Clinical Institute of Clinical Chemistry and Biochemistry, Ljubljana, Slovenia; 4 National Food Administration, Toxicology Division, Uppsala, Sweden; 5 International Centre for Diarrhoeal Disease Research, Bangladesh, Dhaka, Bangladesh; 6 San Antonio de Los Cobres Hospital, San Antonio de Los Cobres, Argentina

**Keywords:** arsenic, *AS3MT*, *BHMT*, *DNMT1a*, *DNMT3b*, gene expression, methylation, one-carbon metabolism, PEMT, polymorphism

## Abstract

**Background:**

Arsenic (As) occurs as monomethylarsonic acid (MMA) and dimethylarsinic acid (DMA) in humans, and the methylation pattern demonstrates large interindividual differences. The fraction of urinary MMA is a marker for susceptibility to As-related diseases.

**Objectives:**

We evaluated the impact of polymorphisms in five methyltransferase genes on As metabolism in two populations, one in South America and one in Southeast Asia. The methyltransferase genes were arsenic(+III oxidation state) methyltransferase (*AS3MT*), DNA-methyltransferase 1a and 3b (*DNMT1a* and *DNMT3b*, respectively), phosphatidylethanolamine N-methyltransferase (*PEMT*), and betaine-homocysteine methyltransferase (*BHMT*). *AS3MT* expression was analyzed in peripheral blood.

**Methods:**

Subjects were women exposed to As in drinking water in the Argentinean Andes [*n* = 172; median total urinary As (U-As), 200 μg/L] and in rural Bangladesh (*n* = 361; U-As, 100 μg/L; all in early pregnancy). Urinary As metabolites were measured by high-pressure liquid chromatography/inductively coupled plasma mass spectrometry. Polymorphisms (*n* = 22) were genotyped with Sequenom, and *AS3MT* expression was measured by quantitative real-time polymerase chain reaction using TaqMan expression assays.

**Results:**

Six *AS3MT* polymorphisms were significantly associated with As metabolite patterns in both populations (*p* ≤ 0.01). The most frequent *AS3MT* haplotype in Bangladesh was associated with a higher percentage of MMA (%MMA), and the most frequent haplotype in Argentina was associated with a lower %MMA and a higher percentage of DMA. Four polymorphisms in the DNMT genes were associated with metabolite patterns in Bangladesh. Noncoding *AS3MT* polymorphisms affected gene expression of *AS3MT* in peripheral blood, demonstrating that one functional impact of *AS3MT* polymorphisms may be altered levels of gene expression.

**Conclusions:**

Polymorphisms in *AS3MT* significantly predicted As metabolism across these two very different populations, suggesting that *AS3MT* may have an impact on As metabolite patterns in populations worldwide.

Millions of people around the world are exposed to high concentrations of arsenic (As) in drinking water, and these exposures are associated with a number of adverse health effects (International Agency for Research on Cancer 2004; National Research Council 2001). There is a marked individual variability in As metabolism, as measured by the distribution of inorganic As (iAs) and the main methylated metabolites monomethylarsonic acid (MMA) and dimethylarsinic acid (DMA) in urine (Vahter 2002). Several studies have shown an association between an increased fraction of MMA in urine, probably reflecting the highly toxic MMA^III^ in the tissues, and an increased risk of various As-related adverse health effects (Chen et al. 2005; Chung et al. 2009; Lindberg et al. 2008; Tseng 2007). As is metabolized by a series of reduction and methylation reactions. *S*-Adenosyl methionine (SAM) is the main methyl donor, implying that As methylation is dependent on factors influencing one-carbon metabolism (Vahter 2002). As(+III oxidation state) methyltransferase (AS3MT) methylates As (Lin et al. 2002). We previously reported an unusual metabolism of As in a mainly indigenous population in the Argentinean Andes, with a lower percentage of MMA and a higher percentage of DMA compared with most other populations (Vahter et al. 1995). This is thought to be partly due to their high frequencies of *AS3MT* alleles associated with a lower percentage of MMA (%MMA) and higher percentage of DMA (%DMA) (Schläwicke Engström et al. 2007, 2009). The importance of *AS3MT* polymorphisms for As metabolism has also been also emphasized in other studies, both for noncoding variants (Agusa et al. 2009; Meza et al. 2005; Valenzuela et al. 2009) and for the exonic variant Met287Thr (Hernandez et al. 2008; Lindberg et al. 2007b; Valenzuela et al. 2009). However, there are few data on influential genetic variants in Bangladesh, where about 50 million people drink water with elevated As concentrations and where the inhabitants have shown an efficient methylation of As to DMA, despite prevalent malnutrition, which is likely to impair one-carbon metabolism (Li et al. 2008). Functional data for *AS3MT* polymorphisms are scarce, apart from a few studies on the Met287Thr variant (Drobna et al. 2004; Wood et al. 2006). Polymorphisms in the promoter and intronic regions may affect the expression of the *AS3MT* gene, which in turn is likely to affect the metabolite pattern.

The methylation of As may partly be an AS3MT-independent process, because methylated As species have been found in *As3mt*-knockout mice, albeit a lower fraction than found in *As3mt* wild-type mice (Drobna et al. 2009). This may be explained by an As-methylating capacity of other methyltransferases. Such candidates could be the DNA methyltransferases (DNMT1a, DNMT3b). DNMTs and AS3MT use SAM as a methyl donor, and there are several parallel characteristics of As methylation and DNA methylation (Vahter 2007).

Several different enzymes are involved in the one-carbon metabolism that generates SAM. For example, the phosphatidylethanolamine *N*-methyltransferase (PEMT) and betaine-homocysteine methyltransferase (BHMT) may influence As metabolism either by regulating the efficiency of the one carbon metabolism or by direct methylation of As.

In this study, we evaluated the impact of polymorphisms in the above-mentioned methyltransferases (*AS3MT*, *DNMT1a*, *DNMT3b*, *PEMT*, and *BHMT*) on As metabolism in two different female populations: in the Andean part of northern Argentina and in rural Bangladesh. Both populations are known to have a wide range of exposure to As in drinking water (Concha et al. 2006; Schläwicke Engström et al. 2007; Vahter et al. 2006), and both populations show efficient methylation of As (Li et al. 2008; Vahter et al. 1995). Furthermore, we investigated the association between *AS3MT* polymorphisms and *AS3MT* gene expression in peripheral blood (Argentina), and the association between *AS3MT* gene expression and As metabolite pattern.

## Materials and Methods

### Study areas and populations

The study participants in Argentina were mainly women from San Antonio de los Cobres (SAC), a village in the northern Argentinean Andes. All subjects in SAC consumed drinking water from the same source; this water contained about 200 μg As/L, with small variations over time (Concha et al. 2006). To achieve a wider range of exposure, we also included in the study women from three nearby villages: Tolar Grande (3.5 μg As/L in drinking water; *n* = 21), Olacapato (12 μg As/L; *n* = 9), and Salar de Pocitos (73 μg As/L; *n* = 5). The study subjects in these three villages were very similar to the study subjects from SAC regarding ethnicity and socioeconomic factors. In total, urine and blood samples were collected from 176 women (not first-degree relatives) in 2008 (172 of which had both metabolite and genotyping data). The samples did not overlap with the women from the same region included in our earlier studies (Schläwicke Engström et al. 2007, 2009).

The Bangladeshi women studied were from a longitudinal mother–child study of As-related health effects in Matlab, a rural area 53 km southeast of Dhaka. This study group is part of a large, population-based, randomized food and micronutrient supplementation trial in pregnancy (Maternal and Infant Nutrition Interventions in Matlab MINIMat). The study group and sampling procedures were described in detail by Li et al. (2008) and Vahter et al. (2006). Urine and blood were sampled in early pregnancy, urine during gestational week 8, and blood during gestational week 14, on average. From the 2,119 women who were enrolled during 2002, 500 women were randomly chosen for the present study. In total, 440 of the 500 women remained in the trial to receive micronutrient supplementation (no supplementation had been received before the sampling of urine), and 408 of those women provided a blood sample. Of these, urine for gestational week 8 was available for 361 individuals.

The Ethical Review Committee of the International Centre for Diarrhoeal Disease Research, Bangladesh; the Health Ministry of Salta (Argentina); and the Ethics Committee of the Karolinska Institutet (Sweden) approved this study. Oral and written informed consent regarding sample collection was obtained from all participants in Argentina and Bangladesh. The results of the present study were evaluated blind for the individuals’ identity, and none of the participants will receive feedback regarding their own results.

### Assessment of As exposure

Exposure to iAs was assessed by the total concentration of As metabolites in urine (U-As; sum of iAs, MMA, and DMA). Speciation of As metabolites in urine was performed using high-pressure liquid chromatography (HPLC) hyphenated with hydride generation and inductively coupled plasma mass spectrometry (ICPMS) (Agilent 1100 series system, Agilent 7500ce; Agilent Technologies, Tokyo, Japan, and Waldbronn, Germany), employing adequate quality control (Lindberg et al. 2007a; Schläwicke Engström et al. 2007). As concentrations were adjusted to the mean specific gravity for each population (1.020 g/mL in Argentina and 1.012 g/mL in Bangladesh), measured by a hand refractometer (Atago, Tokyo, Japan).

### Genotyping

DNA was isolated from peripheral blood samples from both groups of subjects, and genotyping was performed by Swegene’s DNA Facility at Malmö University Hospital (Malmö, Sweden) using Sequenom technology (San Diego, CA, USA). Genotyped polymorphisms (22 polymorphisms in five methyltransferases) are shown in Supplemental Material, Table 1 (doi:10.1289/ehp.1002471).

### Gene expression

We analyzed expression of the *AS3MT* gene (which covers 11 exons) by quantitative real-time polymerase chain reaction (qPCR) assays in peripheral blood samples from participants in Argentina. The rationale for the gene expression analyses was to evaluate whether the *AS3MT* polymorphisms affected gene expression, and if their possible effect on gene expression was due to alternative splicing of *AS3MT*. To evaluate potential alternative splicing, we ran qPCR assays for four different gene segments. If there was an alternative splicing of the gene, we assumed that the polymorphism that was closest to the amplified region was more likely to have an effect on the splicing of the nearby segments than those situated farther from the amplified region. The assays covered segments near the polymorphisms rs3740400 [assay 1: Taqman assay Hs00960536_g1, exons 1–2; polymorphism numbers from the National Center for Biotechnology Information (NCBI 2010b)], rs3740393 (assay 2: Hs00960529_m1, exons 5–6), and rs3740390 (assay 3: Hs00960532_g1, exons 8–9; all from Applied Biosystems, Foster City, CA, USA) and polymorphism rs1046778 [assay 4: 3′ untranslated region, base pairs 37,166–37,264 in AY817668 (NCBI 2010a)], using Applied Biosystems’ Assays-by-Design. We also evaluated whether *AS3MT* polymorphisms were associated with the expression of cyclin M2 (*CNNM2*; Hs00929648_m1, exons 1–2), which is located 16,500 base pairs from the *AS3MT* 3′ end, with rs1046778 as the closest polymorphism.

For each gene expression assay, we tested associations with the genotype of the closest polymorphism in subsets of the total population that included approximately equal numbers of women with each of the three possible genotypes for that polymorphism [*n* per assay = 27–29; matched on a group level for age, weight, body mass index (BMI), U-As, and %MMA]. We repeated the analysis of assay 4 on a larger number of subjects with samples available, matched for age, weight, BMI, and U-As (*n* = 55). Peripheral blood was collected in PAX tubes (PreAnalytiX GmbH, Hombrechtikon, Switzerland), and all samples were frozen and stored at −20°C, after no more than 24 hr at room temperature after sampling. RNA was extracted with the PAXgene Blood RNA Kit (PreAnalytiX) and stored at −80°C. RNA concentration and purity were evaluated on a NanoDrop spectrophotometer (Thermo Scientific, Wilmington, DE, USA; for all samples, 260/280 nm mean optical density ratio = 2.3; range, 1.7–3.3), and RNA integrity (RIN) was evaluated on a Bioanalyzer 2100 (Agilent, Santa Clara, CA, USA; for 17 samples, mean RIN = 8.5; range, 7.6–9.1). For reverse transcription, we used the High Capacity RNA-to-cDNA Kit (Applied Biosystems). Preamplification for *AS3MT* was performed before qPCR using TaqMan PreAmp MasterMix (Applied Biosystems). *AS3MT* expression was normalized to hypoxanthine phosphoribosyltransferase 1 (*HPRT1*) and TATA-binding protein (*TBP*). *HPRT1* and *TBP* were quantified using SYBR Green PCR Master Mix (Applied Biosystems) in nonpreamplified cDNA. The qPCR (20 μL reaction) was run under standard conditions on an ABI7900 instrument (Applied Biosystems) and performed in triplicate with a negative control included for each assay. Samples with an SD < 0.2 for the threshold cycle (C_T_) value were accepted. C_T_ values of each sample were normalized to the mean C_T_ value of each reference gene (ΔC_T_ = AS3MT C_T_ − reference gene C_T_). The ΔC_T_ values were converted to a linear form using the term 2^−ΔCT^. The coefficients of variance for the mean C_T_ values (2^−CT^) for all samples were 22% for *HPRT1* and 32% for *TBP,* and we found no difference in expression between individuals with high and low U-As. We reran assay 4 for the 55 individuals to evaluate repeatability. The Pearson product-moment correlation coefficient (*r*) for the two runs was 0.99.

### Statistical analyses

The study groups from Argentina and Bangladesh were analyzed separately. Deviations from Hardy-Weinberg equilibrium were tested using chi-square analysis. We performed linkage disequilibrium (LD) analysis using Haploview (Barrett et al. 2005). Haplotypes were inferred using PHASE software (Stephens and Donnelly 2003).

In consideration of normally distributed residuals, the percentage of iAs (%iAs), %MMA, and total U-As were natural log (ln)-transformed for both study populations in all analyses. Associations between genotypes or haplotypes (independent variables) and urinary As metabolites (dependent variables: %iAs, %MMA, and %DMA) were analyzed by one-way analysis of variance. For each metabolite (dependent variables), we used a multivariable regression model (using the general linear model) that included genotype or haplotype (as categorical variables) and ln(U-As) (as a continuous and independent variable) to derive *p*-values for associations of genotypes or haplotypes with each metabolite. Additionally, for each metabolite, we used a multivariable regression model that included genotype or haplotype, ln(U-As), and adjustments for other variables. For each metabolite, all potentially influential independent variables were tested in univariate analyses, and those with *p* < 0.2 were included in the latter multivariable regression model. The potentially influential variables tested for the Argentinean study group were body weight, BMI, age, parity (all modeled as continuous variables), and coca use (yes/no), whereas those for the Bangladeshi study group were gestational week, body weight, BMI, age, and parity (all modeled as continuous variables).

Each polymorphism was modeled as a categorical variable (zero, one, or two alleles). When the frequency of a homozygous genotype was very low (one to three individuals), the group was pooled with the heterozygotes. Haplotypes were also modeled as categorical variables (zero, one, or two copies of the haplotype). The genotype or haplotype associated with a low %MMA and a high %DMA was used as the reference genotype, because the aim was to discover which genotypes are associated with high fractions of MMA, a risk factor for As-related health effects. Observed geometric or arithmetic mean percent metabolite values are reported according to genotype for polymorphisms that were significantly associated with one or more metabolites (*p* < 0.05) in models adjusted for ln(U-As) only or in models adjusted for ln(U-As) and other variables. Observed geometric or arithmetic mean percent metabolite values are also reported for the most common *AS3MT* haplotypes. We used models for hypothesis testing only, and no model-based estimates are reported.

We evaluated associations of *AS3MT* genotype with *AS3MT* gene expression using the nonparametric Kruskal-Wallis test. The association between gene expression in assay 4 and As the metabolite pattern in urine was evaluated using Spearman’s rank correlation coefficient (*r*_s_).

All statistical analyses were performed using SPSS (version 17; SPSS Inc., Chicago, IL, USA). We considered *p* < 0.05 to be statistically significant.

## Results

### General characteristics

All polymorphisms were in Hardy-Weinberg equilibrium except for *DNMT1a* rs2228611 in Argentina (χ^2^ = 4.9, *p* = 0.03; in Bangladesh, χ^2^ = 0.4, *p* 0.52).

Descriptive data for both study groups are presented in Table 1. No smokers were included in the study groups. Compared with women in Bangladesh, women in Argentina had U-As about twice as high (median, 200 μg/L; Bangladesh median, 100), lower %MMA (7.9% vs. 10%), and higher %DMA (80 vs. 75%). The U-As range was wide in both populations (10–1,200 in Argentina; 5–1,200 in Bangladesh).

### *AS3MT* genotype analysis

The seven noncoding *AS3MT* polymorphisms had completely different allelic frequencies in the two study groups [see Supplemental Material, Table 1 (doi:10.1289/ehp.1002471)]. In Argentina, all *AS3MT* polymorphisms, except for rs11191439 (Met287Thr), showed strong LD (*R*^2^ between 0.82 and 0.98; see Supplemental Material, Figure 1). In Bangladesh, we detected two LD clusters: one cluster with rs3740400, rs10748835, and rs1046778 (cluster 1) and one cluster with rs3740393, rs3740390, and rs11191453 (cluster # 2) (see Supplemental Material, Figure 1). The polymorphism rs11191439 showed no LD with any of the other *AS3MT* polymorphisms evaluated in either study population.

In Argentina, all *AS3MT* polymorphisms, except for the nonsynonymous rs11191439, were significantly associated with the metabolite pattern, and for each polymorphism the allele associated with lower %iAs, lower %MMA, and higher %DMA was the most common (Table 2). In Bangladesh, all *AS3MT* polymorphisms except rs7085104 were significantly associated with the metabolite pattern. However, patterns of associations varied between the two LD clusters: Polymorphisms in cluster 1 were significantly associated with %MMA but not %iAs or %DMA, whereas polymorphisms in cluster 2 were significantly associated with %iAs and %DMA but not %MMA. In most cases, the alleles associated with lower %MMA or lower %iAs and higher %DMA were the less frequent ones in Bangladesh.

In both populations, participants possessing one or two copies of haplotype 1 had higher %MMA than did those who did not carry this haplotype (Table 3). Haplotype 1 was the most common haplotype in Bangladesh (52%). Haplotype 2 was associated with lower %iAs, lower %MMA, and higher %DMA in both populations (although %MMA was not statistically significant in Bangladesh) and was more frequent in Argentina (70%) than in Bangladesh (16.5%). Haplotype 3 was significantly associated with lower %MMA, but this haplotype was present almost exclusively in Bangladesh (16.5%). Haplotype 4 was associated with higher %iAs and lower %DMA in both populations, although this was statistically significant only in Bangladesh. This was the only haplotype containing the C-allele of rs11191439, and we found no individuals with two copies of this haplotype in either population.

### *AS3MT* expression analysis

**Figure 1:**
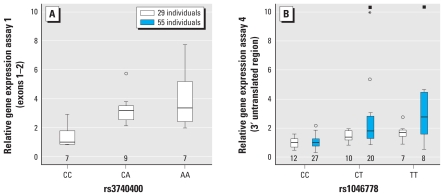
Box plots depicting relative *AS3MT* gene expression according to genotype in women from Argentina; expression for the reference genotype (associated with low %MMA and high %DMA) is set to 1. (*A*) Relative gene expression based on assay 1 (covering exons 1–2) according to rs3740400 genotype. (*B*) Relative gene expression based on assay 4 (covering part of the 3′ untranslated region) according to rs1046778 genotype. Numbers below the boxes indicate the number of individuals with that genotype. The bottom and top of each box indicate 25th and 75th percentiles, respectively; the line inside the box is the median; whiskers represent the smallest and the largest values that are not outliers. Circles and asterisks indicate outliers; squares indicate outliers outside the range of the *y*-axis, which had a gene expression of 25 (CT genotype) and 23 (TT genotype).

Heterozygous and homozygous genotypes that included alleles associated with high %MMA and low %DMA among the women in Argentina were associated with higher *AS3MT* expression than were corresponding reference genotypes associated with low %MMA and high %DMA. Associations were statistically significant for rs3740400 based on assay 1 (observed gene expressions for the AA and CA genotypes were 240% and 220% higher, respectively, than for the CC genotype; *p* = 0.009) and for rs1046778 based on assay 4 (observed gene expressions for the TT and CT genotypes were 69% and 36%, respectively, higher than for the CC genotype; *p* = 0.008) (Figure 1); the *p*-values for the other assays were 0.18 (for rs3740393 based on assay 2) and 0.077 (for rs3740390 based on assay 3). We ran both endogenous control genes for all assays. The results were very similar when using *HPRT1* or *TBP* as endogenous controls, but the *HPRT1* gene demonstrated less variability (the coefficient of variance was 22% for *HPRT1* vs. 32% for *TBP*). We found no association between the *AS3MT* rs1046778 polymorphism and expression of the downstream gene *CNNM2* (*p* = 0.82). Carriers of each genotype demonstrated a similar expression pattern for all *AS3MT* gene segments analyzed, and we found no evidence that a certain genotype caused alternative splicing. We repeated the gene expression analysis based on assay 4 on a larger set of individuals (*n* = 55) and observed the same pattern: relative to the rs1046778 CC genotype, *AS3MT* expression was 175% and 80% higher among those with rs1046778 TT and CT genotypes, respectively (*p* < 0.001; Figure 1). Among these 55 individuals, expression of *AS3MT* (assay 4) was positively (but not significantly) associated with %iAs (*r*_s_ = 0.16, *p* = 0.24) and %MMA (*r*_s_ = 0.18, *p* = 0.18) and inversely associated with %DMA (*r*_s_ = −0.28, *p* = 0.090).

### DNMT1a and DNMT3b.

The minor allele frequencies for the five polymorphisms analyzed in *DNMT1a* were 4–40% in Argentina and 2–44% in Bangladesh, whereas the minor allele frequencies for the three polymorphisms analyzed in *DNMT3b* were 5–6% in Argentina and 23–44% in Bangladesh [see Supplemental Material, Table 1 (doi:10.1289/ehp.1002471)]. The LD pattern for the DNMT genes was similar in Bangladesh and Argentina. The rs10854076 and rs2228612 polymorphisms in *DNMT1a* showed strong LD in both populations (*R*^2^ = 0.99 in Argentina, 0.84 in Bangladesh), whereas the *R*^2^ values between the other polymorphisms in *DNMT1a* were rather low. All *DNMT3b* polymorphisms showed LD in both study populations, with *R*^2^ values ranging from 0.39 to 0.79 in Bangladesh and from 0.88 to 1 in Argentina.

We found no significant associations between DNMT polymorphisms and As metabolism among the Argentinean women. However, four DNMT polymorphisms were significantly associated with metabolite patterns among women in Bangladesh (Table 2): The *DNMT1a* polymorphisms rs2228612 and rs10845076 (in complete LD) predicted %iAs (*p* = 0.028, highest for the common rs2228612 AA variant), whereas the *DNMT3b* polymorphisms rs6087990 and rs2424913 predicted %iAs (*p* = 0.030 and 0.044, highest for the common rs6087990 CC and rs2424913 TT genotypes, respectively) and %DMA (*p*-values = 0.013 and 0.021, respectively, with lower mean values for the CC and TT genotypes). We observed no significant associations with metabolite patterns in either population for *DNMT1a* rs2228611, rs16999593, or rs7253062 or for *DNMT3b* rs2424932 (data not shown).

### PEMT and BHMT.

For *PEMT*, rs1531100 and rs4244598 showed LD for both populations (*R*^2^ = 0.70 in Bangladesh and 1 in Argentina), but rs2278952 and rs897453 showed no LD with any other *PEMT* polymorphism evaluated. The two *BHMT* polymorphisms showed no LD. Polymorphisms in *PEMT* or *BHMT* were not statistically significant predictors of As metabolite patterns.

## Discussion

This study demonstrated strong associations between *AS3MT* polymorphisms and As metabolism in two populations, one from the Argentinean Andes and one from Bangladesh, both of which are heterogeneous in genetic background and in living conditions. The finding of two LD clusters with different patterns of metabolite fractions suggests the presence of several influential polymorphisms along the gene. Noncoding polymorphisms in *AS3MT* were significantly associated with altered gene expression. In addition, four polymorphisms in *DNMT1a* and *DNMT3b* were associated with As metabolites among the pregnant women from Bangladesh.

One strength of this study is that both populations had wide ranges of both total U-As and As metabolites. Also, the study subjects within each population were rather homogeneous regarding social status and food habits. However, the populations differed from each other in a number of ways, not only genetically but also regarding food habits, nutritional status, lifestyle factors, and pregnancy (all women from Bangladesh were in early pregnancy). This strengthens the hypothesis that the impact of *AS3MT* on As metabolite pattern should be valid for people worldwide and not strongly modified by environmental or biological factors.

We are aware that relatively few individuals were included in the gene expression analyses. Nevertheless, genotype was highly predictive of the gene expression of *AS3MT*. This was apparent despite the fact that gene expression was measured in peripheral blood, which has a lower expression of *AS3MT* than in the main As-methylating tissues, that is, liver and kidney (Su et al. 2004). We used blood as a proxy for liver because sampling liver tissues is a difficult and invasive procedure. It is, however, important to note that extrapolation of gene expression from blood to liver is difficult because of tissue-specific differences in transcription factors and other regulating elements, as well as differences in metabolite distributions between blood and liver, that may affect the result.

Six polymorphisms were associated with As metabolite pattern in subjects from both Argentina and Bangladesh. Several *AS3MT* polymorphisms were associated with %iAs, which we did not detect in our earlier studies in Argentina (Schläwicke Engström et al. 2007, 2009), possibly due to a lower power in those studies (which included about 100 fewer individuals). We confirmed associations between six *AS3MT* polymorphisms and As metabolism observed in our earlier studies in another group of individuals from the same region in Argentina (Schläwicke Engström et al. 2007, 2009). Additionally, two polymorphisms not investigated in any of our earlier studies (rs11191453 and rs1046778) significantly predicted the metabolite pattern. Because many polymorphisms showed strong LD and because the significance levels are dependent on the genotype frequencies, it is not possible to attribute causal effects to specific variants. However, in Bangladesh we observed associations with %MMA mainly for polymorphisms in cluster 1 (rs3740400, rs10748835, and rs1046778), which are situated in the 5′ and the 3′ end of the gene, whereas we observed associations with %iAs and %DMA mainly for polymorphisms in cluster 2 (rs3740393, rs3740390, and rs11191453), which are situated in the middle and the 3′ end of the gene. We confirmed previous findings of a higher %MMA and lower %DMA in association with the Thr allele in Met287Thr (rs111191439) (Hernandez et al. 2008; Lindberg et al. 2007b; Valenzuela et al. 2009). The Thr allele in Met287Thr has also been associated with a marginally higher risk (*p* = 0.055) of premalignant skin lesions (Valenzuela et al. 2009). This polymorphism had rather low frequencies in the populations studied, which were from Chile (Hernandez et al. 2008), Eastern Europe (Lindberg et al. 2007b), and Mexico (Valenzuela et al. 2009), where the allele frequencies were around 5–10%.

The fact that the women from Bangladesh were in early pregnancy may have had an impact on the association between polymorphisms in methyltransferases and As metabolite patterns. Pregnancy is known to induce methylation reactions required for tissue growth and development, as well as As methylation (Concha et al. 1998; Szyf 2009), which may have obscured associations with polymorphisms in methyltransferase genes among the women in Bangladesh. However, to determine the effect of pregnancy on the associations between genotype and As metabolite pattern, pregnant and nonpregnant women from the same population should be compared, or a study with repeated sampling during pregnancy could be performed.

The frequencies of the *AS3MT* polymorphisms associated with As metabolism deviated markedly between the Argentinean and Bangladeshi populations. In the Argentinean population, the alleles and haplotypes associated with more efficient methylation were very frequent, which is interesting considering the fact that the indigenous people have been living in this As-contaminated area for many generations (Figueroa et al. 1992), suggesting a selection for genotypes associated with efficient As metabolism. In Bangladesh, where most of the tube wells have been installed in the last 20–40 years, the alleles associated with more efficient methylation were the least common; nevertheless, allele frequencies were rather high (~ 45%) for the polymorphisms in LD cluster 1. Still, the general evidence of associations between *AS3MT* genotype on the As metabolite fraction was similar in both populations. The present study, as well as other studies (Agusa et al. 2009; Hernandez et al. 2008; Lindberg et al. 2007b; Meza et al. 2005), strongly support a causal association of *AS3MT* genotype on As metabolism worldwide.

A higher %MMA is linked to several adverse health effects (Hsueh et al. 1997; Lindberg et al. 2008; Tseng 2007; Yu et al. 2000). Individuals in Bangladesh who had an MMA level > 8% had a significantly higher risk (*p* < 0.01) for skin lesions compared with the reference group (where %MMA was < 8) (Lindberg et al. 2008). As demonstrated here, *AS3MT* genotypes significantly predict the fraction of metabolites and may explain part of the individual differences in the fraction of MMA detected and, in turn, explain differences in susceptibility to skin lesions. Thus, *AS3MT* genotype is probably a susceptibility factor for As toxicity, and the allelic distribution of *AS3MT* polymorphisms should be taken into account in future risk evaluations of As in drinking water and food.

In the present study, we demonstrate for the first time that noncoding *AS3MT* polymorphisms were associated with *AS3MT* gene expression levels. The common homozygotes in the Argentinean population were associated with lower %MMA and higher %DMA and were also associated with lower *AS3MT* expression relative to other genotypes. We could not determine whether associations were causal effects due to a single *AS3MT* polymorphism or a specific haplotype, because all polymorphisms, spread throughout the gene, showed strong LD in Argentina. However, polymorphisms in the 5′ part of the gene (assay 1, 5′ of rs3740400) and 3′ untranslated region (assay 4, including rs1046778), which are likely to involve regulatory sequences, were significantly associated with gene expression. In addition, expression was also associated, although not significantly, with metabolite patterns in urine. We saw no indications that the genotype influenced the splicing of the gene, because we observed a similar pattern of gene expression for all four gene segments analyzed.

Wood et al. (2006) evaluated the potential impact of polymorphisms on *AS3MT* protein levels *in vitro*, where the transient expression of the wild-type *AS3MT* sequence and the variant allozyme of Met287Thr were evaluated in COS-1 monkey kidney cells. Endogenous activity of untransfected COS-1 cells was negligible. The 287Thr allozyme, which has been associated with a higher %MMA and lower %DMA, had higher *in vitro* protein levels than did those of the wild type. In the present study, very few Argentinean subjects possessed the Thr allele, so it was not possible to evaluate whether this polymorphism was associated with *AS3MT* gene expression. However, for the polymorphisms that we included in the gene expression analyses, the alleles associated with higher %MMA and lower %DMA were associated with higher gene expression, similar to the association between the 287Thr allele, linked to higher %MMA and lower %DMA, and higher *in vitro* protein levels. Gene expression and protein levels are not always correlated because posttranscriptional regulation processes may be involved. Further studies are needed to evaluate the impact of polymorphisms on the protein level.

We observed associations with DNMT polymorphisms in Bangladesh only, and these results need to be confirmed. *DNMT1a* rs16999593 was previously associated with lower %DMA in Argentina (Schläwicke Engström et al. 2009), but this association was not evident in the present study population. However, some of the DNMT polymorphisms had a very low allele frequency in Argentina.

## Conclusions

Polymorphisms in *AS3MT* significantly predicted As metabolism in these two very different populations, which suggests that an effect of *AS3MT* on As metabolite pattern may be present in populations worldwide. *AS3MT* polymorphisms were also associated with *AS3MT* expression in peripheral blood, such that polymorphisms associated with efficient methylation were associated with reduced expression of the *AS3MT* gene. Evidence of an effect of the polymorphisms is of importance for risk assessment of As, because a low methylation efficiency is associated with increased risk for As toxicity.

## Figures and Tables

**Table 1:** Descriptive data for the study populations.

	Argentina			Bangladesh		
Variable	*n*	Median	90th–10th percentile	*n*	Median	90th/10th percentile
U-As (μg/L)^a^	172	200	27–450	361	100	21–390
iAs (%)	172	12	4.9–21	361	14	7–24
MMA (%)	172	7.9	4.6–12	361	10	5–17
DMA (%)	172	80	69–89	361	75	62–85
Age (years)	172	34	20–58	361	26	19–35
Weight (kg)	172	57	46–75	359	44	37–53
Height (cm)	172	152	145–159	361	150	144–158
BMI (kg/m^2^)	172	24	20–32	359	19	17–24
Parity	168	3	1–8	360	1	0–4
Coca use (%)	172	46		NA		

NA, not applicable.

a Adjusted for specific weight.

**Table 2:** Observed mean values^a^ for As metabolites according to genotype for polymorphisms that were significant predictors of As metabolites in the regression models.

Gene/rs number, genotype^b^	Argentina				Bangladesh			
	*n*	%iAs	%MMA	%DMA	*n*	%iAs	%MMA	%DMA
*AS3MT*								

rs7085104								

GG	93	9.6	7.1	81.2	30	13.7	9.7	74.5
GA	62	11.7	8.6	77.6	147	13.7	10.2	73.5
AA	15	14.2	10.3	73.5	182	13.4	9.5	74.3
*p*-Value 1^c^		0.03	0.001	0.001		0.89	0.14	0.58
*p*-Value 2^d^		0.051	0.002	0.002		0.85	0.14	0.58

rs3740400^e^								

CC	93	9.6	7.1	81.2	71	14.1	8.8	74
CA	61	11.8	8.5	77.5	180	13.6	10.2	73.7
AA	15	14.2	10.3	73.5	103	13.3	9.7	74.6
*p*-Value 1		0.027	0.001	< 0.001		0.74	0.008	0.51
*p*-Value 2		0.058	0.003	0.003		0.73	0.008	0.51

rs3740393^f^								

CC	85	9.2	6.9	81.9	13	10.8	7.9	79.8
GC	68	12	8.7	77.2	105	12	9.2	76.8
GG	17	14.7	10	73.3	240	14.5	10.1	72.5
*p*-Value 1		0.003	< 0.001	< 0.001		0.003	0.13	0.001
*p*-Value 2		0.007	0.001	< 0.001		0.003	0.13	0.001

rs3740390^f^								

AA	84	9.3	6.9	81.8	11	11.3	7.8	79.4
AG	69	11.9	8.7	77.4	94	12.2	9.4	76.5
GG	17	14.7	10	73.2	255	14.2	10	72.9
*p*-Value 1		0.006	< 0.001	< 0.001		0.025	0.15	0.004
*p*-Value 2		0.018	0.001	< 0.001		0.034	0.15	0.005

rs11191439								

MetMet	163	10.7	7.8	79.4	313	13.2	9.7	74.6
MetThr	6	12.6	10.2	75.5	42	16.7	11	69
*p*-Value 1		0.86	0.22	0.45		0.004	0.086	< 0.001
*p*-Value 2		0.67	0.1	0.26		0.005	0.086	0.001

rs11191453^f^								

CC	83	9.3	6.9	81.9	11	11.3	7.8	79.4
CT	71	12	8.7	77.3	93	12.2	9.6	76.3
TT	17	14.7	10	73.3	250	14.3	10	72.8
*p*-Value 1		0.004	< 0.001	< 0.001		0.015	0.17	0.004
*p*-Value 2		0.007	< 0.001	< 0.001		0.015	0.17	0.005

rs10748835^e^								

AA	93	9.6	7.1	81.3	67	14.4	8.5	73.9
AG	62	12	8.6	77.3	189	13.2	10.2	74.2
GG	15	14.2	10.3	73.5	95	14.1	10	73.4
*p*-Value 1		0.014	0.001	< 0.001		0.38	0.001	0.94
*p*-Value 2		0.035	0.001	0.002		0.39	0.001	0.97

rs1046778^e^								

CC	87	9.3	6.9	81.9	45	13.1	7.9	76.5
CT	69	12.1	8.7	77.2	183	13.1	9.6	74.6
TT	15	14.2	10.3	73.5	132	14.3	10.7	72.4
*p*-Value 1		0.005	< 0.001	< 0.001		0.38	< 0.001	0.052
*p*-Value 2		0.013	< 0.001	< 0.001		0.4	< 0.001	0.083

*DNMT1a*								

rs2228612 (in LD with rs10845076)								

GG	21	12.7	7.5	77.1	20	11.9	9.9	76.7
GA	62	9.8	7.8	80.4	123	13	9.7	74.6
AA	88	11	8	78.9	217	14	9.8	73.5
*p*-Value 1		0.13	0.88	0.17		0.17	0.89	0.42
*p*-Value 2		0.17	0.9	0.23		0.028	0.89	0.4

*DNMT3b*								

rs6087990								

TT	—				34	13.7	10.6	73.8
TC	19	12.1	8.6	76.9	145	12.5	9.8	75.8
CC	150	10.5	7.8	79.6	180	14.5	9.7	72.6
*p*-Value 1		0.12	0.12	0.059		0.015	0.33	0.007
*p*-Value 2		0.32	0.099	0.12		0.030	0.33	0.013

rs2424913								

CC	—				26	12.5	10.2	75.5
CT	17	11.7	8	78	133	12.6	9.8	75.6
TT	153	10.6	7.8	79.4	200	14.3	9.7	72.8
*p*-Value 1		0.24	0.49	0.25		0.037	0.64	0.020
*p*-Value 2		0.45	0.48	0.42		0.044	0.64	0.021

a %iAs and %MMA are both geometric means, whereas the %DMA is an arithmetic mean.

b The *AS3MT* genotype associated with a low %MMA and a high %DMA in Argentina is listed first.

c *p*-Value 1 for genotype coefficients (coded as categorical variables according to the number of variant alleles) from multivariate regression models for each population and outcome [ln(%iAs), ln(%MMA), or %DMA in Argentina or Bangladesh] adjusted for ln(U-As) only; the models were not used to derive mean values for the metabolites.

d *p*-Value 2 for genotype coefficients (coded as categorical variables) from multivariate regression models for each location and outcome with adjustment for ln(U-As) and the following covariates: for Argentina ln(%iAs): coca use, age, parity; Argentina ln(%MMA): coca use, BMI, parity; Argentina %DMA: coca use, BMI, age, parity; Bangladesh ln(%iAs) and %DMA: age, BMI. Models used to test associations with ln(%MMA) in Bangladesh were adjusted for ln(U-As) only because none of the other variables tested had a *p* < 0.2 in the univariate model. Models were used for hypothesis testing only and were not used to derive mean values for the metabolites.

e Polymorphisms in *AS3MT* LD cluster # 1 in Bangladesh.

f Polymorphisms in LD cluster # 2.

**Table 3:** Observed percentages of As metabolites according to copy number (zero, one, or two) of the common *AS3MT* haplotypes.

	Argentina				Bangladesh			
Haplotype/sequence^a^	0 copies	1 copy	2 copies	*p*-Value^b^	0 copies	1 copy	2 copies	*p*-Value^b^
1. AAGGTTGT								

*n* (%)	97 (57)	60 (35)	15 (9)		79 (22)	192 (53)	90 (25)	
Geometric mean %iAs	9.5	11.9	14.2	0.015	14.1	13.2	14.0	0.66
Geometric mean %MMA	7.1	8.6	10.3	0.001	8.7	10.2	10.0	0.006
Mean %DMA	81.4	77.5	73.5	< 0.001	74.1	74.2	73.6	0.97

2. GCCATCAC								

*n* (%)	17 (10)	71 (41)	84 (49)		255 (71)	95 (26)	11 (3)	
Geometric mean %iAs	14.7	12.0	9.0	0.001	14.2	12.2	11.3	0.027
Geometric mean %MMA	10.0	8.7	6.8	< 0.001	10.0	9.5	7.8	0.16
Mean %DMA	73.3	77.3	82.2	< 0.001	72.9	76.4	79.4	0.005

3. ACGGTTAC								

*n* (%)	171 (99.4)	1 (0.6)	—		250 (69)	104 (29)	7 (2)	
Geometric mean %iAs	NA	NA	—	NA	13.3	14.3	11.4	0.16
Geometric mean %MMA	NA	NA	—	NA	10.3	8.7	8.0	< 0.001
Mean %DMA	NA	NA	—	NA	74.2	73.4	78.7	0.16

4. GCGGCTAT								

*n* (%)	166 (96)	6 (4)	—		322 (89)	39 (11)	—	
Geometric mean %iAs	10.7	12.6	—	0.27	13.2	17.1	—	0.002
Geometric mean %MMA	7.7	10.2	—	0.27	9.6	11.1	—	0.088
Mean %DMA	79.4	75.4	—	0.45	74.7	68.2	—	< 0.001

Abbreviations: *n,* number of individuals; NA, not applicable (no or very few individuals with that haplotype).

a Polymorphisms are listed in 5′ to 3′ direction: rs7085104, rs3740400, rs3740393, rs3740390, rs11191439, rs11191453, rs10748835, and rs1046778. Only haplotypes with a frequency > 5% in any population are included; for individuals with missing data for one or more polymorphisms, the missing alleles were inferred using PHASE.

b *p*-Values for haplotype coefficients (coded as categorical variables according to the number of copies of the haplotype) from regression models for each population and outcome [ln(%iAs), ln(%MMA), or %DMA in Argentina or Bangladesh] adjusted for ln(U-As) only. The models were not used to derive mean values for the metabolite; models with adjustments for other influential variables (see information for *p*-value 2 Table 2 footnote *d*) were also evaluated, but the *p*-values were very similar when adjusting for ln(U-As) only (data not shown).

## References

[b1-ehp-119-182] Agusa T, Iwata H, Fujihara J, Kunito T, Takeshita H, Minh TB (2009). Genetic polymorphisms in AS3MT and arsenic metabolism in residents of the Red River Delta, Vietnam. Toxicol Appl Pharmacol.

[b2-ehp-119-182] Barrett JC, Fry B, Maller J, Daly MJ (2005). Haploview: analysis and visualization of LD and haplotype maps. Bioinformatics.

[b3-ehp-119-182] Chen CJ, Hsu LI, Wang CH, Shih WL, Hsu YH, Tseng MP (2005). Biomarkers of exposure, effect, and susceptibility of arsenic-induced health hazards in Taiwan. Toxicol Appl Pharmacol.

[b4-ehp-119-182] Chung WH, Sung BH, Kim SS, Rhim H, Kuh HJ (2009). Synergistic interaction between tetra-arsenic oxide and paclitaxel in human cancer cells in vitro. Int J Oncol.

[b5-ehp-119-182] Concha G, Nermell B, Vahter M (2006). Spatial and temporal variations in arsenic exposure via drinking-water in northern Argentina. J Health Popul Nutr.

[b6-ehp-119-182] Concha G, Vogler G, Lezcano D, Nermell B, Vahter M (1998). Exposure to inorganic arsenic metabolites during early human development. Toxicol Sci.

[b7-ehp-119-182] Drobna Z, Naranmandura H, Kubachka KM, Edwards BC, Herbin-Davis K, Styblo M (2009). Disruption of the arsenic (+3 oxidation state) methyltransferase gene in the mouse alters the phenotype for methylation of arsenic and affects distribution and retention of orally administered arsenate. Chem Res Toxicol.

[b8-ehp-119-182] Drobna Z, Waters SB, Walton FS, LeCluyse EL, Thomas DJ, Styblo M (2004). Interindividual variation in the metabolism of arsenic in cultured primary human hepatocytes. Toxicol Appl Pharmacol.

[b9-ehp-119-182] Figueroa LT, Razmilic BB, González MU, Sancha AM (1992). Corporal distribution of arsenic in mummied bodies owned to an arsenical habitat. International Seminar Proceedings: Arsenic in the Environment and Its Incidence on Health.

[b10-ehp-119-182] Hernandez A, Xamena N, Surralles J, Sekaran C, Tokunaga H, Quinteros D (2008). Role of the Met(287)Thr polymorphism in the AS3MT gene on the metabolic arsenic profile. Mutat Res.

[b11-ehp-119-182] Hsueh YM, Chiou HY, Huang YL, Wu WL, Huang CC, Yang MH (1997). Serum beta-carotene level, arsenic methylation capability, and incidence of skin cancer. Cancer Epidemiol Biomarkers Prev.

[b12-ehp-119-182] International Agency for Research on Cancer (2004). Some drinking-water disinfectants and contaminants, including arsenic. IARC Monog Eval Carcinog Risk Hum.

[b13-ehp-119-182] Li L, Ekstrom EC, Goessler W, Lonnerdal B, Nermell B, Yunus M (2008). Nutritional status has marginal influence on the metabolism of inorganic arsenic in pregnant Bangladeshi women. Environ Health Perspect.

[b14-ehp-119-182] Lin S, Shi Q, Nix FB, Styblo M, Beck MA, Herbin-Davis KM (2002). A novel *S*-adenosyl-l-methionine:arsenic(III) methyltransferase from rat liver cytosol. J Biol Chem.

[b15-ehp-119-182] Lindberg AL, Goessler W, Grander M, Nermell B, Vahter M (2007a). Evaluation of the three most commonly used analytical methods for determination of inorganic arsenic and its metabolites in urine. Toxicol Lett.

[b16-ehp-119-182] Lindberg AL, Kumar R, Goessler W, Thirumaran R, Gurzau E, Koppova K (2007b). Metabolism of low-dose inorganic arsenic in a central European population: influence of sex and genetic polymorphisms. Environ Health Perspect.

[b17-ehp-119-182] Lindberg AL, Rahman M, Persson LA, Vahter M (2008). The risk of arsenic induced skin lesions in Bangladeshi men and women is affected by arsenic metabolism and the age at first exposure. Toxicol Appl Pharmacol.

[b18-ehp-119-182] Meza MM, Yu L, Rodriguez YY, Guild M, Thompson D, Gandolfi AJ (2005). Developmentally restricted genetic determinants of human arsenic metabolism: association between urinary methylated arsenic and CYT19 polymorphisms in children. Environ Health Perspect.

[b19-ehp-119-182] NCBI (National Center for Biotechnology Information) (2010a). Nucleotide Database.

[b20-ehp-119-182] NCBI (National Center for Biotechnology Information) (2010b). Single Nucleotide Polymorphism Database.

[b21-ehp-119-182] National Research Council (2001). Arsenic in Drinking Water: 2001 Update.

[b22-ehp-119-182] Schläwicke Engström K, Broberg K, Concha G, Nermell B, Warholm M, Vahter M (2007). Genetic polymorphisms influencing arsenic metabolism: evidence from Argentina. Environ Health Perspect.

[b23-ehp-119-182] Schläwicke Engström K, Nermell B, Concha G, Stromberg U, Vahter M, Broberg K (2009). Arsenic metabolism is influenced by polymorphisms in genes involved in one-carbon metabolism and reduction reactions. Mutat Res.

[b24-ehp-119-182] Stephens M, Donnelly P (2003). A comparison of Bayesian methods for haplotype reconstruction from population genotype data. Am J Hum Genet.

[b25-ehp-119-182] Su AI, Wiltshire T, Batalov S, Lapp H, Ching KA, Block D (2004). A gene atlas of the mouse and human protein-encoding transcriptomes. Proc Natl Acad Sci USA.

[b26-ehp-119-182] Szyf M (2009). The early life environment and the epigenome. Biochim Biophys Acta.

[b27-ehp-119-182] Tseng CH (2007). Arsenic methylation, urinary arsenic metabolites and human diseases: current perspective. J Environ Sci Health C Environ Carcinog Ecotoxicol Rev.

[b28-ehp-119-182] Vahter M (2002). Mechanisms of arsenic biotransformation. Toxicology.

[b29-ehp-119-182] Vahter ME (2007). Interactions between arsenic-induced toxicity and nutrition in early life. J Nutr.

[b30-ehp-119-182] Vahter M, Concha G, Nermell B, Nilsson R, Dulout F, Natarajan AT (1995). A unique metabolism of inorganic arsenic in native Andean women. Eur J Pharmacol.

[b31-ehp-119-182] Vahter ME, Li L, Nermell B, Rahman A, El Arifeen S, Rahman M (2006). Arsenic exposure in pregnancy: a population-based study in Matlab, Bangladesh. J Health Popul Nutr.

[b32-ehp-119-182] Valenzuela OL, Drobná Z, Hernández-Castellanos E, Sánchez-Peña LC, Garcia-Vargas GG, Borja-Aburto VH (2009). Association of AS3MT polymorphisms and the risk of premalignant arsenic skin lesions. Toxicol Appl Pharmacol.

[b33-ehp-119-182] Wood TC, Salavagionne OE, Mukherjee B, Wang L, Klumpp AF, Thomae BA (2006). Human arsenic methyltransferase (AS3MT) pharmacogenetics: gene resequencing and functional genomics studies. J Biol Chem.

[b34-ehp-119-182] Yu RC, Hsu KH, Chen CJ, Froines JR (2000). Arsenic methylation capacity and skin cancer. Cancer Epidemiol Biomarkers Prev.

